# Carboplatin dose capping affects pCR rate in HER2-positive breast cancer patients treated with neoadjuvant Docetaxel, Carboplatin, Trastuzumab, Pertuzumab (TCHP)

**DOI:** 10.1007/s10549-020-05868-z

**Published:** 2020-08-29

**Authors:** Sacha J. Howell, Faye Coe, Xin Wang, Laura Horsley, Maria Ekholm

**Affiliations:** 1 Department of Medical Oncology, The Christie NHS Foundation Trust, Manchester, UK; 2 Division of Cancer Sciences, University of Manchester, Manchester, UK; 3 Department of Pharmacy, The Christie NHS Foundation Trust, Manchester, UK; 4 Department of Analytics and Statistics, Digital Services, The Christie NHS Foundation Trust, Manchester, UK; 5 Department of Oncology, Ryhov Hospital, 551 85 Jönköping, Sweden; 6 Department of Clinical Sciences, Division of Oncology and Pathology, Lund University, Lund, Sweden

**Keywords:** Carboplatin, Administration and dosing, Breast neoplasms, Neoadjuvant therapy, Treatment outcome

## Abstract

**Purpose:**

Estimated glomerular filtration rate (eGFR) is commonly used to calculate carboplatin doses and capping the eGFR may be used to reduce the risk of excessive dosing and toxicity. We sought to retrospectively examine the impact of our carboplatin guidelines on pathological complete response rates (pCR) and toxicity in women with HER2+ breast cancer receiving neoadjuvant docetaxel, carboplatin, trastuzumab and pertuzumab (TCHP).

**Methods:**

The delivered area under the curve (dAUC) was calculated [(actual carboplatin dose at cycle 1 ÷ dose calculated with uncapped/unbanded eGFR) × 6] and dichotomized at the median value. The impact of this and other clinical factors on pCR rate, dose intensity (DI) and toxicity was assessed.

**Results:**

124 eligible patients were identified of whom 63.7% (79/124) achieved pCR. The median dAUC at cycle 1 was 5.75 mg × ml/min. Those with lower dAUC were more frequently younger and overweight/obese. Patients with lower dAUC had significantly inferior pCR rates of 54.8% (34/62) vs 72.6% (45/62), respectively (*p* = 0.040). Similar results were seen in the ER+ subgroup; 45.2% (19/42) vs 68.3% (28/41), *p* = 0.037%, whereas no significant difference was seen among ER- patients; 75.0% (15/20) vs 81.0% (17/21), *p* = 0.72. DI and toxicity were comparable between the two dAUC groups.

**Conclusions:**

The overall pCR rate was high in patients with HER2+ breast cancer receiving the TCHP regimen; however, carboplatin dose capping resulted in inferior pCR rates, particularly in the ER+ subgroup. To ensure optimal dosing, isotopic measurement of renal function is warranted in patients who would otherwise have their eGFR and dose capped.

## Introduction

**Fig. 1:**
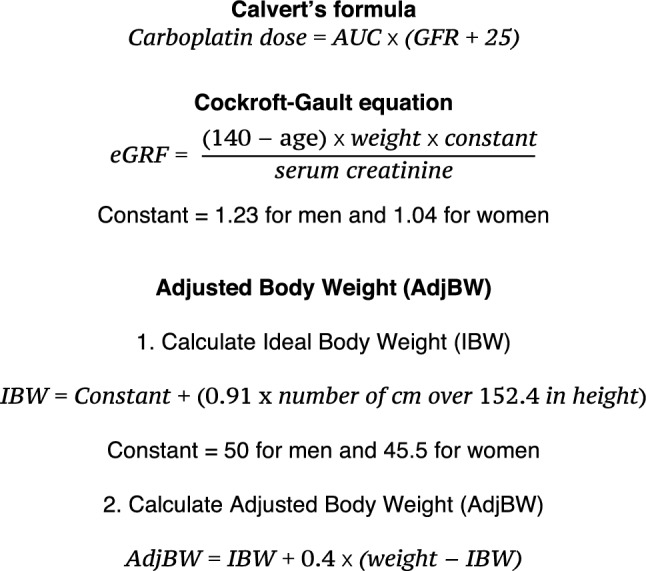
The Calvert formula, the Cockroft-Gault formula and the formula for calculating Adjusted Body Weight

Renal function is linearly related to carboplatin plasma clearance and the dose is usually calculated with the Calvert formula (Fig. 1) [1]. However, the optimal strategy for measurement of the glomerular filtration rate (GFR) and thus for dosing carboplatin is the subject of much debate [2–5]. GFR is most accurately measured (mGFR) using isotopic methods such as 51Cr-EDTA [6], but such assays are often impractical. Multiple formulae to estimate GFR (eGFR) have been developed, predominantly in non-cancer patient populations [3, 7–10]. These formulae frequently result in significant measurement error, compared with isotopic mGFR, particularly at the extremes of body weight [3, 4]. Body surface area (BSA) corrected formulae or the use of adjusted body weight (AdjBW) (Fig. 1) in those with raised body mass index (BMI), for example in the commonly used Cockroft-Gault (CG) formula (Fig. 1) [7], have been shown to improve eGFR correlation with mGFR [11, 12]. In contrast, use of the Wright formula (without weight correction) to calculate eGFR in women with ovarian cancer has shown inferior survival outcomes and reduced toxicity, indicating suboptimal dosing in obese patients [13]. Carboplatin pharmacokinetics are clearly modified by obesity and the optimal dosing strategy is unclear.

The introduction of Isotope Dilution Mass Spectrometry (IDMS) to standardise serum creatinine (SCr) measurement, which gives lower SCr levels and thus higher eGFR [14, 15], has led to widespread capping of eGFR (most commonly at 125 ml/min) to reduce the risk of severe toxicity [16]. Capping and correction for raised BMI are primarily designed to reduce toxicity, however there is also the potential for under-dosing, resulting in suboptimal treatment effects. Two retrospective studies have examined capped vs uncapped approaches and neither reported increased haematological toxicity in patients dosed according to uncapped eGFR [17, 18].

At The Christie NHS Foundation Trust (The Christie), docetaxel (T) carboplatin (C) trastuzumab (H) pertuzumab (P) (TCHP) is a commonly used neoadjuvant regimen for patients with human epidermal growth factor receptor 2 (HER2)-positive breast cancer. Carboplatin is dosed on actual body weight (ABW) irrespective of BMI and eGFR capped at 110 ml/min. The efficacy of neoadjuvant therapy can be objectively judged by the pathological complete response (pCR) rate, which has been shown in meta-analyses to predict for long-term survival outcomes [19, 20]. We conducted a retrospective review of cases treated with neoadjuvant TCHP to determine the effect of carboplatin dosing on pCR rates.

## Methods

The Christie electronic prescribing databases were used to identify patients treated with TCHP in the neoadjuvant setting. Medical records were reviewed for patient and tumour characteristics. ER positivity was defined as ≥ 1% of cells staining positively [21]. T stage was recorded as the maximum tumour diameter on pretreatment magnetic resonance imaging (MRI), or ultrasound/mammogram or clinical examination if no MRI was performed. For patients with multifocal breast cancer the size of the largest tumour was used. pCR was defined as the absence of invasive tumour cells in the breast and axillary lymph nodes, i.e. ypT0/TisypN0.

Standard dose TCHP consisted of docetaxel 75 mg/m^2^, carboplatin AUC6, trastuzumab 8 mg/kg at cycle 1 and 6 mg/kg thereafter and pertuzumab 840 mg at cycle 1 and 420 mg thereafter, all given intravenously every 3 weeks for 6 cycles. ABW was used to calculate eGFR using the CG formula [7]. The eGFR was capped at 110 ml/min to avoid excess toxicity. In the UK, ‘dose banding’ is routinely performed to reduce drug wastage based on vial size and results in maximum variance of 6% from the prescribed dose [22–24]. Capping at 110 ml/min resulted in a carboplatin dose of 810 mg, although this was further reduced to 790 mg as a result of ‘dose banding’. G-CSF was used routinely.

### Ethics

The project was approved by The Christie NHS Foundation Trust as a service evaluation (IRB reference number 2541). Service evaluation in England is exempt from ethics committee review (Health Research Authority Guidance; www.HRA.NHS.UK).

### Statistical analyses

Dose capping, ‘dose banding’ and dose reductions can all lead to an AUC that is lower than the intended target. To be able to investigate this further we defined the variable ‘delivered AUC’ (dAUC) for carboplatin as: the delivered dose at cycle 1÷ full dose (according to an uncapped/’unbanded’ eGFR from the CG formula using IDMS SCr and ABW) × 6. For example, for a patient with an eGFR of 135 ml/min the full carboplatin dose would have been 960 mg according to the Calvert formula [(135 + 25) × 6], whereas with capping at 110 ml/min the dose was reduced to 810 mg [(110 + 25) × 6] and ‘dose banding’ further reduced the dose to 790 mg. The dAUC was thus (790 ÷ 960) × 6 = 4.94 mg × ml/min. To determine the effect of dAUC on outcome the data were dichotomized at the median value of 5.75.

Chi-Squared Test or Fisher’s Exact Test (when frequency counts smaller than 5) were applied to assess the association between capping and patient and tumour characteristics and Median Test was applied to compare the median values. Mann–Whitney *U* Test was applied to assess whether population distributions of ‘Delivered carboplatin dose at cycle 1′ differ between the two dAUC groups. Univariable logistic regression were applied to assess the relationship between pCR and age, BMI, ER status, tumour size, N status and dAUC. Odds ratios together with their corresponding 95% confidence interval and Wald P values were calculated. All presented P values are two-sided. Several factors included in the univariable analysis, such as age, weight/BMI and dAUC, are interrelated and therefore multivariable analysis was not performed. Following the univariable analysis, a post hoc exploratory analysis to investigate pCR rates in subgroups were carried out; dAUC (< vs ≥ the median value), BMI (< 25/ ≥ 25), and ER status (pos/neg). Dose intensity (DI), accounting for dose reductions and dose deferrals, was calculated as described by Hryniuk [25, 26]. The occurrence of the greatest thrombocytopenia grade per patient (CTCAE v5.0) was recorded and analysed by groups was also assessed. Statistical analyses were performed using SPSS v26.0 statistical software (SPSS Inc., Chicago, IL, USA).

## Results

### Patients

**Fig. 2:**
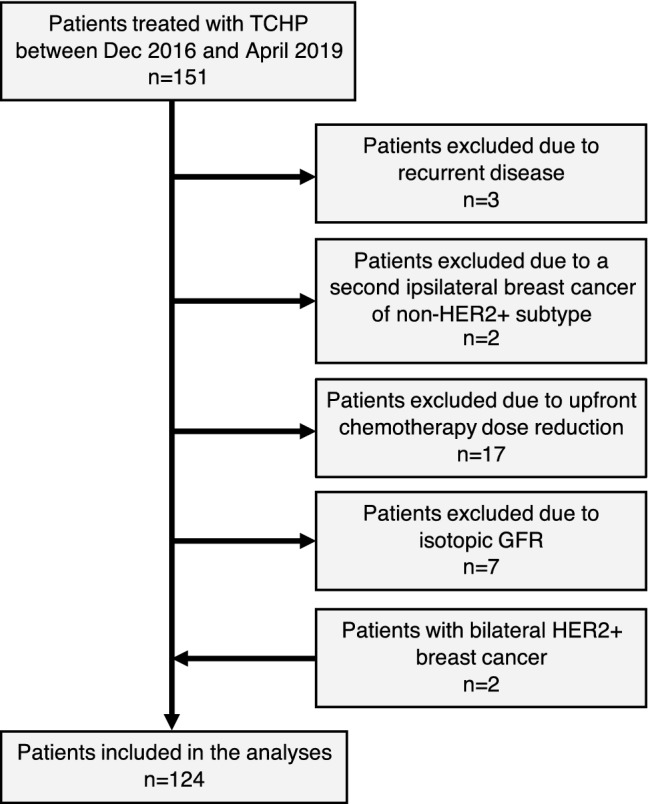
CONSORT diagram illustrating the patient numbers including the reasons for exclusion. *TCHP* docetaxel + carboplatin + trastuzumab + pertuzumab, *HER2* human epidermal growth factor receptor 2, *GFR* glomerular filtration rate

We identified 151 patients treated with TCHP at The Christie between Dec 2016 and April 2019. Seventeen patients with dose reductions of docetaxel (*n* = 13) and/or carboplatin (*n* = 16) at cycle 1, were excluded. In addition, patients with local recurrence (*n* = 3), a second ipsilateral BC of non-HER2 + subtype (*n* = 2) or mGFR based carboplatin dosing (*n* = 7) were excluded. Two patients had bilateral HER2+ cancers which were considered separately (2 tumours per patient) giving a total of 124 patient tumours (hereafter referred to as patients) (Fig. 2 and Table 1). Staging was performed by MRI in 102 patients, mammogram or ultrasound in 20 patients and 2 patients with locally advanced disease had T size measured by clinical examination.

**Table 1:** Patient and tumour characteristics for all patients divided by dAUC group

	All	dAUC < 5.75	dAUC ≥ 5.75	*p* value*
Number of patients	124	62	62	
Age, years				
Median	48	46	50	0.031
Range	24–67	26–59	24–67	
< 40	26 (21.0)	20 (32.3)	6 (9.7)	0.002
≥ 40	98 (79.0)	42 (67.7)	56 (90.3)	
Performance status				1.0
0	115 (95.0)	58 (95.1)	57 (95.0)	
1	6 (5.0)	3 (4.9)	3 (5.0)	
Missing	3	1	2	
BMI, kg/m^2^				< 0.001
Median	26.4	29.0	24.2	
Range	18.0–58.2	20.6–58.2	18.0–36.8	
< 25	51 (41.1)	17 (27.4)	34 (54.8)	< 0.001
≥ 25–30	37 (29.8)	16 (25.8)	21 (33.9)	
≥ 30	36 (29.0)	29 (46.8)	7 (11.3)	
Tumour size, mm				0.18
0–20	14 (11.3)	4 (6.5)	10 (16.1)	
21–50	81 (65.3)	41 (66.1)	40 (64.5)	
≥ 50	29 (23.4)	17 (27.4)	12 (19.4)	
Multifocality				0.58
No	79 (63.7)	41 (66.1)	38 (61.3)	
Yes	45 (36.3)	21 (33.9)	24 (38.7)	
Nodal status				0.47
Negative	54 (43.5)	25 (40.3)	29 (46.8)	
Positive	70 (56.5)	37 (59.7)	33 (53.2)	
ER status				0.85
Positive	83 (66.9)	42 (67.7)	41 (66.1)	
Negative	41 (33.1)	20 (32.3)	21 (33.9)	

*BMI* body mass index, *dAUC* delivered area under the curve, *ER* oestrogen receptor

*Median test, Chi-squared test or Fisher’s exact test

Patients with dAUC < 5.75 were significantly younger and more likely to be overweight/obese (Table 1). This was expected as both factors increase the calculated eGFR, thereby resulting in a greater propensity for dose capping. There were no significant differences between the dAUC groups otherwise in terms of patient and tumour characteristics (Table 1).

### Factors prognostic for pCR

The overall pCR rate was 63.7% (79/124). In univariable analysis dAUC < 5.75, BMI ≥ 25 and ER positivity (ER+) showed significant negative associations with pCR rate (Table 2). The impact of dAUC remained significant in the ER+ subgroup but not in the ER− (Table 3). BMI ≥ 25 was associated with lower pCR rates in the whole cohort and in the ER+, but not significantly in the ER- subgroup (Table 3). pCR was not significantly associated with patient age, pretreatment tumour size or nodal status. In patients with uncapped eGFR (> 110 ml/min) ‘dose banding’ alone did not have a significant effect on pCR rates (data not shown).

**Table 2:** Univariable analysis of association between different factors and pCR (*n* = 124)

	Odds ratio (OR)	95% Confidence interval (CI)	*p *value
Age, years			
≥ 40	Ref.		
< 40	0.49	0.20–1.17	0.11
BMI, kg/m^2^			
< 25	Ref.		
≥ 25	0.37	0.17–0.83	0.015
As continuous variable	0.93	0.87–0.99	0.033
ER status			
Negative	Ref.		
Positive	0.37	0.16–0.87	0.022
Tumour size, mm			
0–20	3.72	0.78–17.75	0.099
21–50	Ref.		
≥ 50	0.88	0.37–2.08	0.77
Nodal status			
cN0	Ref.		
cN+	1.06	0.51–2.21	0.88
dAUC			
≥ 5.75	Ref.		
< 5.75	0.46	0.22–0.97	0.042
As continuous variable	1.73	0.99–3.03	0.056

*BMI* body mass index, *pCR* pathological complete response, *dAUC*, delivered area under the curve, *ER*, oestrogen receptor

**Table 3:** pCR for all patients and divided by dAUC group and further by ER status and BMI

	Number of patients	Absolute difference (%)	*p* value*	dAUC < 5.75	dAUC ≥ 5.75	Absolute difference (%)	*p *value*
All patients							
Number of patients	124			62	62		
pCR, *n* (%)	79 (63.7)			34 (54.8)	45 (72.6)	17.8	0.040
BMI < 25, *n*	51			17	34		
pCR, *n* (%)	39 (76.5)	21.7	0.014	12 (70.6)	27 (79.4)	8.8	0.50
BMI ≥ 25, *n*	73			45	28		
pCR, *n* (%)	40 (54.8)			22 (48.9)	18 (64.3)	15.4	0.20

ER+ tumours							
Number of patients	83			42	41		
pCR, *n* (%)	47 (56.6)			19 (45.2)	28 (68.3)	23.1	0.034
BMI < 25, *n*	31			10	21		
pCR, *n* (%)	22 (71.0)	22.9	0.042	6 (60.0)	16 (76.2)	16.2	0.42
BMI ≥ 25, *n*	52			32	20		
pCR, *n* (%)	25 (48.1)			13 (40.6)	12 (60.0)	19.4	0.17

ER− tumours							
Number of patients	41			20	21		
pCR, *n* (%)	32 (78.0)			15 (75.0)	17 (81.0)	6.0	0.72
BMI < 25, *n*	20			7	13		
pCR, *n* (%)	17 (85.0)	13.6	0.45	6 (85.7)	11 (84.6)	− 0.9	1.0
BMI ≥ 25, *n*	21			13	8		
pCR, *n* (%)	15 (71.4)			9 (69.2)	6 (75.0)	5.8	1.0

*BMI* body mass index, *dAUC* delivered area under the curve, *ER* oestrogen receptor, *pCR* pathological complete response

*Chi-squared test or Fisher’s exact test

### Dosing, Dose Intensity and Toxicity

Neither median DI nor those achieving DI ≥ 85% differed significantly between those receiving dAUC < 5.75 vs dAUC ≥ 5.75. Similarly, there was no significant difference between the frequency of highest grade thrombocytopenia between the groups (Table 4). In exploratory analysis including women with BMI ≥ 25 and an uncapped eGFR (*n* = 29), the use of AdjBW to calculate eGFR in the CG formula would have meant their received dose was effectively at a median dAUC of 7.0 (IQR 6.8 to 7.2). Despite this the proportion achieving > 85% DI (79.3%; 23/29), completing all 6 cycles of treatment (82.8%; 24/29) and experiencing grade 3/4 thrombocytopenia (10.3%; 3/29) was similar to the cohort as a whole (Table 4).

**Table 4:** Dosing, dose intensity and toxicity for all patients and divided by dAUC group

Number of patients	All	dAUC < 5.75	dAUC ≥ 5.75	*p *value*
	124	62	62	
Uncapped eGFR, ml/min				
Median (range)	109 (52–234)	127 (97–234)	90 (52–112)	< 0.001
IQR	89–127	120–142	81–103	
≤ 110	64 (51.6)	3 (4.8)	61 (98.4)	< 0.001
> 110–125	27 (21.8)	26 (41.9)	1 (1.6)	
> 125	33 (26.6)	33 (53.2)	0	
Calculated uncapped/’unbanded’ carboplatin dose, mg				
Median (range)	804 (462–1554)	909 (732–1554)	688 (462–822)	< 0.001
IQR	686–911	870–999	634–770	
Delivered carboplatin dose at cycle 1 (after capping and ‘dose banding’), mg				
Median (range)	790 (450–790)	790 (700–790)	700 (450–790)	< 0.001
IQR	700 –790	790–790	630–790	
Total delivered carboplatin dose, mg				
Median (range)	4180 (560–4740)	4650 (790–4740)	3780 (560–4740)	< 0.001
IQR	3518–4740	4095–4740	3063–4200	
Median number of cycles (range)	5.5 (1–6)	5.6 (1–6)	5.5 (1–6)	0.63
Completed 6 cycles TCHP				0.80
Yes	105 (84.7)	53 (85.5)	52 (83.9)	
No	19 (15.3)	9 (14.5)	10 (16.1)	
DI chemotherapy				
Median (IQR)	93.8 (86.3–100.0)	93.7 (86.8–100.0)	93.9 (83.8–100.0)	1.0
≥ 85%	97 (78.2)	51 (82.3)	46 (74.2)	0.28
< 85%	27 (21.8)	11 (17.7)	16 (25.8)	
dAUC				
Median (range)	5.75 (3.05–6.36)	5.21 (3.05–5.74)	6.01 (5.77–6.36)	< 0.001
IQR	5.20–6.02	4.74–5.45	5.89–6.12	
Thrombocytopenia				
Grade 0	47 (37.9)	25 (40.3)	22 (35.5)	0.84
Grade 1	54 (43.5)	25 (40.3)	29 (46.8)	
Grade 2	10 (8.1)	5 (8.1)	5 (8.1)	
Grade 3	9 (7.3)	4 (6.5)	5 (8.1)	
Grade 4	4 (3.2)	3 (4.8)	1 (1.6)	

*dAUC* delivered area under the curve, *DI* dose intensity, *eGFR* estimated glomerular filtration rate, *IQR* inter quartile range, *TCHP* docetaxel + carboplatin + trastuzumab + pertuzumab

*Median test, Chi-squared test, Fisher’s exact test or Mann–Whitney *U* test

## Discussion

The data presented demonstrate that carboplatin dose capping at 110 ml/min results in inferior pCR rates in a cohort of women with early HER2+ breast cancer, receiving potentially curative neoadjuvant therapy. The pCR rates with neoadjuvant TCHP of 64% overall and 57% in the ER+ cohort are comparable to the rates of 66% and 50% (total) and 56% and 44% (ER+) reported in the TRYPHAENA and KRISTINE studies respectively, using the same regimen [27, 28]. These comparable pCR rates were achieved despite capping the eGFR at 110 ml/min, which in combination with ‘dose banding’ resulted in a maximum carboplatin dose of 790 mg. The KRISTINE study protocol recommended following FDA guidance and capping the eGFR at 125 ml/min if IDMS SCr methodology was used and using ‘an appropriate method as per their routine practice’ for calculating eGFR in patients with high BSA. It is not possible, therefore, to determine exactly how eGFR was calculated for the 221 patients that received TCHP in the KRISTINE study or the impact of dose capping on the pCR rate [28]. Possibly, AdjBW can have been used, resulting in approximately 1 AUC lower carboplatin dose in comparison to when using ABW, potentially partially explaining the difference in pCR rates and frequency of grade 3/4 thrombocytopenia; 1.8% in comparison to 10.5% and 11.5% reported in the current study and in TRYPHAENA, respectively [27, 28]. The methodology for eGFR calculation in the smaller TRYPHAENA study is not published [27]. Carboplatin dose capping is designed to reduce excessive chemotherapy toxicity from spuriously high, non-physiological eGFR calculations. In the cohort of women receiving lower than average dAUC, the proportion achieving > 85% DI (i.e. having fewer dose reductions and dose delays) was numerically higher than in the above average dAUC group, without evidence of increased toxicity. In the face of significantly inferior pCR rates this suggests that the carboplatin dose may be suboptimal in this cohort.

In the current study the reduced dAUC appeared to be of particular importance in the ER+ subgroup in which the pCR rate was 23 percentage points greater in women with dAUC ≥ 5.75 vs dAUC < 5.75 (68% vs 45%, *p* = 0.034). In comparison, in the ER− subgroup, where the pCR rates were higher overall, the 6% point increase in pCR rate with above median dAUC was not statistically significant. Although the numbers in this subgroup are small and the study retrospective, this observation is concordant with previous data showing that ER−/HER2+ cancers, on average, have greater HER2 amplification/expression and a greater dependency on HER2 signalling [29]. Indeed pCR with anti-HER2 therapy alone is seen more commonly in ER−/HER2+ tumours [30], suggesting dAUC may be less important in this subgroup.

Our results show that it is possible to achieve high pCR rates using the TCHP regimen, provided that the carboplatin is adequately dosed. As carboplatin pharmacokinetics are linearly related to renal function, accurate assessment of GFR is vital. The CG formula has been shown to result in mean/median absolute percent error in carboplatin dose of 10–14% [18, 31], and more than a quarter of patients have a carboplatin dose absolute percentage error of at least 20% [4]. In potentially curative disease settings, errors resulting in inappropriate under-dosing are of particular concern. However, overweight/obese patients are more prone to overestimation of eGFR and, potentially, excess toxicity [4, 18]. In a small pharmacokinetic based study in patients with BMI > 27, AdjBW resulted in an achieved mean AUC of 5.7 (SD 1.7) compared to the desired value of 5.2, whereas the estimated AUC using ABW was 6.7 (SD 2.1) [11]. In our cohort, women with BMI ≥ 25 and an uncapped eGFR would have had an effective 1 AUC reduction in carboplatin dose through the use of AdjBW, which would likely have resulted in further reduced pCR rates. Consequently, the use of ABW in our cohort may explain the high pCR rates achieved despite dose capping at 110 ml/min. Renal function generally starts to decline around the age of 30 [32], which means that younger patients are more likely to experience dose capping. The impact of overweight/obesity and age on eGFR were evident in this study as 72.6% and 32.3% of the women in the low dAUC group were overweight/obese or < 40 years, compared with 45.2% and 9.7%, respectively in those in the high dAUC group (Table 1). Although capping at an eGFR of 110 ml/min is lower than some established guidelines, all capping strategies including at the 125 ml/min level should be validated in additional cohorts. More than half of the patients in the dAUC ≤ 5.75 in this study would also have been capped at the 125 ml/min eGFR threshold (Table 4). Until the effect of such dose reduction has been confirmed as safe we suggest mGFR is performed to assess GFR accurately in patients who would otherwise have their carboplatin dose capped. The impact such a change has on toxicity and DI is not known and should be assessed in future studies.

Although the primary aim of the TRYPHAENA trial was to assess the tolerability of different regimens with focus on cardiac safety and the study was not powered to compare outcome, the pCR rate was numerically higher in the TCHP arm (66.2%) than in the two anthracycline containing arms (61.6% and 57.3%) [27]. The TRAIN-2 study compared FEC × 3 → Paclitaxel + C × 6 vs Paclitaxel + C × 9, both with concurrent H + P, and found similar pCR rates (68% vs 67%), but less febrile neutropenia in the non-anthracycline arm [33]. Carboplatin as part of the chemotherapy backbone seems to be an important drug for neoadjuvant treatment of HER2 + BC, particularly for the ER+ subgroup. However, considering the toxicity associated with the TCHP regimen, weekly paclitaxel or nab-paclitaxel in combination with weekly carboplatin (AUC2) + H + P, may be a more tolerable regimen worth investigating in a clinical trial.

As stated above, pCR has been shown to correlate with survival outcomes in HER2+ BC [19, 20]. There is currently a trend in oncology toward de-escalation of therapy. While these attempts to reduce unnecessary treatment and thus toxicity are laudable, there are concerns that outcomes could also suffer. For example, the 5 year follow up from the NeoSPHERE study suggests that disease free survival may be inferior without early use of a regimen containing both cytotoxic and dual HER2 targeting [34]. Although both adjuvant trastuzumab emtansine and neratinib have been shown to improve outcome after completion of neoadjuvant and adjuvant therapy respectively [35, 36], it is not yet known whether this salvage approach is as effective as early optimization of neoadjuvant therapy to maximise the rate of pCR. Furthermore, both treatments are associated with additional costs and in the case of neratinib in particular, significant toxicity. If pCR rates can be augmented through optimization of carboplatin dosing, the need for additional adjuvant therapies could potentially be avoided.

There are several limitations of this study. First it is based on a retrospective cohort at a single institution. Second, capping at an eGFR of 110 ml/min (effectively 107–114 ml/min due to ‘dose banding’) is not a standard approach and the impact of capping at 125 ml/min could not be tested. Third, in common with most studies examining dosing strategies the toxicity data are a surrogate for carboplatin dosing in the absence of pharmacokinetic data.

## Conclusion

Considering the high pCR rates demonstrated in this study, particularly for the ER+/HER2+ subgroup, carboplatin is an important chemotherapy option in the treatment for early HER2+ breast cancer. Moreover, our results caution against the temptation to reduce the carboplatin dose in TCHP to AUC 5 as this is likely to reduce the pCR rate. However, the uncertainties related to calculation of eGFR remain of concern. It is important that oncologists are aware of the pitfalls in carboplatin dosing. Isotopic methods to measure GFR more accurately should be employed more frequently, particularly in patients in whom eGFR would be capped. Consistent guidelines on how to calculate Carboplatin doses are warranted.

## Data Availability

Data are available on request to the corresponding author.
